# A one-year exercise intervention program in pre-pubertal girls does not influence hip structure

**DOI:** 10.1186/1471-2474-9-9

**Published:** 2008-01-24

**Authors:** Gayani Alwis, Christian Linden, Susanna Stenevi-Lundgren, Henrik G Ahlborg, Jack Besjakov, Per Gardsell, Magnus K Karlsson

**Affiliations:** 1 Clinical and Molecular Osteoporosis Research Unit, Department of Clinical Sciences, Lund University, Sweden; 2 Department of Orthopedics, Malmö University Hospital, SE-205 02 Malmö, Sweden

## Abstract

**Background:**

We have previously reported that a one-year school-based exercise intervention program influences the accrual of bone mineral in pre-pubertal girls. This report aims to evaluate if also hip structure is affected, as geometry independent of bone mineral influences fracture risk.

**Methods:**

Fifty-three girls aged 7 – 9 years were included in a curriculum-based exercise intervention program comprising 40 minutes of general physical activity per school day (200 minutes/week). Fifty healthy age-matched girls who participated in the general Swedish physical education curriculum (60 minutes/week) served as controls. The hip was scanned by dual X-ray absorptiometry (DXA) and the hip structural analysis (HSA) software was applied to evaluate bone mineral content (BMC), areal bone mineral density (aBMD), periosteal and endosteal diameter, cortical thickness, cross-sectional moment of inertia (CSMI), section modulus (Z) and cross-sectional area (CSA) of the femoral neck (FN). Annual changes were compared. Group comparisons were done by independent student's *t*-test between means and analyses of covariance (ANCOVA). Pearson's correlation test was used to evaluate associations between activity level and annual changes in FN. All children remained at Tanner stage 1 throughout the study.

**Results:**

No between-group differences were found during the 12 months study period for changes in the FN variables. The total duration of exercise during the year was not correlated with the changes in the FN traits.

**Conclusion:**

Evaluated by the DXA technique and the HSA software, a general one-year school-based exercise program for 7–9-year-old pre-pubertal girls seems not to influence the structure of the hip.

## Background

Physical activity during growth is associated with benefits in bone mineral accrual and possibly bone structure [1-4], a clinically relevant notion, as both traits independently improve bone strength [5]. But, most prospective controlled exercise intervention trials have predominantly focused on the accrual of bone mineral [6-9], a study design that could underestimate the skeletal effects of exercise, as the effect on bone structure is then neglected. That is, also the three-dimensional structure ought to be assessed when evaluating bone strength [10,11]. As a result, the Hip Structural Analysis (HSA) was developed, a software that based on a dual energy X ray absorbtiometry (DXA) hip scan evaluate the geometrical structure of the femoral neck (FN) [12,13]. Exercise studies that have assessed the method have come to contradictory conclusions, some supporting [14,15], other opposing [16,17] that training influence the hip structure. The different inferences could be based on the fact that different intensities and types of training program may affect the skeleton differently. As a result, the current study was designed to evaluate if a moderate intense school curriculum based exercise intervention program, possible for all children to participate within, in a population based cohort of pre-pubertal girls could influence the geometrical structure of FN. It must also be emphasized that the study did not aim to estimate the osteogenic effect of specific activities or separately evaluate more or less active children. Instead we wanted to estimate if physical activity could be used as a population based prevention strategy to influence the structure of the FN.

## Methods

The Malmö Pediatric Osteoporosis Prevention (POP) study was conducted as a prospective controlled investigation of the impact of an exercise intervention on skeletal development in children from school start and onwards. The baseline measurements in the school, that was allocated as intervention school, were made before the intervention was initiated and the follow-up measurements one year later. Three neighboring control schools, allocated within the same city suburb, were evaluated in the same way, but the follow-up measurements of the control group were done in the same months but two years later. Due to lack of resources in our research laboratory, controls were re-measured first after two years. However, we accepted a two-year follow-up for the controls, because data in the literature imply that skeletal growth and accrual of bone mineral is linear in Tanner stage 1 and before the age of ten [3,18-26]. The design of the investigation has previously been evaluated and regarded as accurate when following growth and bone mineral accrual in this age group [8].

The four schools that were contacted agreed to participate. All four were located in the same part of the city in areas that were socio-economically similar; they were government funded; and allocation of pupils to the schools was done according to residential address. Before the intervention, they all used the Swedish standard curriculum for physical education (PE) to the same extent. After the four schools accepted participation, one of them was asked to be the intervention school and agreed to that. The intervention school modified its curriculum by increasing the number of PE classes; in other words, we did not choose a school that already had a high level of physical activity (PA) for its pupils. The other three schools were included as a control cohort. Furthermore, in order to ascertain whether there was any selection bias at baseline, we compared the girls who took part in the study with those who refused to participate. This was done by considering data on height, weight, and body mass index (BMI) obtained from the initial standard compulsory school health evaluation of first grade pupils. The drop-out analyses revealed that, based on data from the school records, there were no significant differences at baseline in height, weight, or BMI between girls who participated in the study and those who refused to take part [8,9].

In the intervention school, all 61 girls in grades 1 and 2 were invited to attend, and 55 agreed to participate (attendance rate 90%). We excluded one girl at baseline, because she was 11 months younger than the second-youngest girl. At follow up, one girl declined further participation. Therefore, a total of 53 girls with a mean age of 7.7 ± 0.6 (mean ± SD), (range 6.5–8.7), years at baseline were included in the intervention group. Sixty-four volunteers participated at baseline as controls. At follow-up, 13 had moved out of the region or declined further participation, and we excluded one girl because she was being treated with growth hormone. Thus a total of 50 girls with a mean age of 7.9 ± 0.6 (range 6.8–8.9) at baseline were included in the control group. All participants were healthy Caucasians who had no diseases and were not taking medications known to influence bone metabolism.

The exercise intervention was initiated at the beginning of the school term after the baseline measurement. The program consisted of both indoor and outdoor physical activities that are ordinarily included in the PE curriculum in Swedish schools, such as running, jumping, climbing ropes, and playing a variety of ball games. The curriculum was supervised by the usual class teacher and was increased from 60 minutes per week to 200 minutes per week (40 minutes/day), thus no specific osteogenic training program was added. Furthermore, the teachers were instructed to vary the activities and sports, so that the children would not get bored by repetition. The aim was to minimize the number of dropouts, which has been reported to be fairly high during and after exercise intervention programs [27]. The control schools continued with the same type of activities as previously used, but the duration was kept within the limits stipulated by the compulsory PE curriculum, consisting of one to two sessions of PE per week, in total 60 minutes per week.

During the dual X-ray absorptiometry (DXA, DPX-L version 1.3z, Lunar^®^, Madison, WI) measurements the children were dressed in light clothes and no shoes. Paediatric software was used in all scans with children below 35 kg in weight. That is, the software was changed in some children between the different measurements. All standard image files of the proximal femur were analyzed by one technician, by use of the hip strength analysis (HSA) software. The software is provided by Lunar Instruments Corporation (Madison, WI). Using this software the X-ray absorption data of the proximal femur is extracted from the output image data file and the bone mineral content (BMC, g) and areal bone mineral density (aBMD, g/cm^2^) and its distribution within the FN calculated. First the operator has to manually define the center of the femoral head and place the FN axis as accurately as possible along the FN. Thereafter, the region of interest in the FN is placed in the proximal part of the FN, and finally the femoral shaft axis is defined centrally along the shaft. The software will then iteratively assess all cross sections in the FN region of interest and identify the plane with the least cross-sectional moment of inertia (CSMI, cm^4^). CSMI is an estimate of the ability of the FN to withstand bending forces, and that value was calculated using the mass distribution derived from the absorption curve [28]. The CSMI estimated by DXA has been found to be highly correlated with the CSMI measured directly on adult cadaver specimens (r^2 ^= 0.96) [28]. Automatic identification of the weakest cross section of the FN is fundamental feature of the HSA software, and this cross-sectional level is then used for subsequent calculation of section modulus (Z, cm^3^) and cross-sectional area (CSA, cm^2^). The section modulus is also an estimate of the ability of the FN to withstand bending forces, and it was computed as CSMI divided by half the width of the FN. CSA is a measure of the resistance of the bone to axial forces, and it represents the area of mineral packed together in the defined cross section of the FN and is essentially proportional to the bone mineral content (BMC). In addition, endosteal diameter was estimated using the algorithm described by Thomas J. Beck [29]. Mean cortical thickness was calculated as the difference between periosteal and endosteal diameter divided by two. The FN vBMD was computed using the formula vBMD = BMC/estimated FN volume (π × r^2 ^× FN length), where r = FN mid-diameter/2, assuming the FN to be cylindrical [30,31].

The coefficient of variation (CV) was evaluated by duplicate measurements in 13 healthy children ages seven to fifteen (mean 10) years was found to be 1.4% for BMC, 1.6% for aBMD, 3.7% for total body fat mass, and 1.5% for total body lean mass. The CV values for the HSA analyses were 1.5% for FN periosteal diameter, 2.2% for FN CSA, and 6.2% for FN CSMI. The machine was calibrated daily with the Lunar^® ^phantom. The technicians in our research group performed all the measurements, and one technician conducted all software analyses. Total lean mass and total fat mass were estimated from the DXA total body scan. Body weight was measured to the nearest 0.1 kg using an electric scale, and height was determined to the nearest 0.5 cm using a wall-mounted measuring rod.

A questionnaire previously used in several studies but modified for pre-pubertal children [32,33] was employed to evaluate lifestyle factors, including the level of physical activity. The total time spent in such activity was calculated as time per week in organized sports within the school curriculum and in organized sports activities outside the school. The maturity of the children was assessed by Tanner staging [34], conducted by our research nurses.

Informed written consent was obtained from parents or guardians of participants prior to the study start. The study was approved by the Ethics Committee of Lund University (LU 453-98; 1998-09-15), Sweden, and conducted according to the Helsinki Declaration of 2000. The Swedish Data Inspection Board approved both the data collection and database.

Research has shown the technical problems when scanning small children based on inconsistency in limb positioning and location of the region of interest [35], we advocated the method introduced by Beck et al. when evaluating FN width [12]. This method excludes biologically unlikely values, 3 standard deviations (SD) above or below the mean. This resulted in exclusion of eight proximal femoral scans in the intervention group and six in the control group. In addition, missing scans or scans with obvious technical errors were found in three hip scans in the intervention group and one in the control group. Data are presented as means ± SD. The annual changes were calculated using the data on the evaluated traits. Student's *t*-test between means was used for group comparisons. Analyses of covariance (ANCOVA) were used to adjust for chronological age at baseline and increment in height and weight in the follow up evaluations to adjust any difference in growth. Pearson's correlation test was applied to correlate the total mean duration of physical activity, calculated as the mean of the total physical activity at baseline and at follow-up, with changes in the bone parameters during the study period. Statistical calculations were performed using Statistica^®^, version 6.1 (StatWin^®^). A p-value < 0.05 was defined as a statistically significant difference. With known annual growth in the evaluated parameters in Swedish children aged 7 years and with a significance level of p < 0.05 this study was powered to detect an annual difference in gain in BMC of 0.102 g/yrs, BMD of 0.019 g/cm^2^/yrs, vBMD of 0.011 g/cm^3^/yrs, neck length of 0.6 mm/yrs, periosteal width of 0.5 mm/yrs, CSA of 0.05 cm^2^/yrs, Z of 0.19 cm^3^/yrs, CSMI of 0.029 cm^4^/yrs, endosteal width of 0.5 mm/yrs and cortical thickness of 0.3 mm/yrs.

## Results

As previously reported, the intervention group and the control group did not at baseline differ with regard to registered lifestyle factors or anthropometric parameters [8]. After onset of the exercise program, the intervention group spent comparatively more time in physical activity in total both at baseline and at follow-up (Table 1). All the participants in the two groups remained at Tanner stage 1 during the study period.

**Table 1:** The duration of physical activity at baseline and at follow-up

	Cases	Controls
Organized physical activity (hours/week)		
**Baseline before intervention**		
Outside the school	0.7 ± 1.2	1.3 ± 1.6
**Baseline after intervention**		
School curriculum	3.3	1.0 **
Outside the school	0.7 ± 1.2	1.3 ± 1.6
Total physical activity	4.0 ± 1.2	2.3 ± 1.6 **
**Follow-up**		
School curriculum	3.3	1.0 **
Outside the school	1.1 ± 1.3	1.9 ± 1.9 *
Total physical activity	4.4 ± 1.3	2.9 ± 1.9 **

Data presented as mean ± SD. *P < 0.05, **P < 0.001

The mean values for the FN at baseline were 6.0% higher for aBMD (p = 0.04) and 7.0% higher for cortical thickness (p = 0.02) in the intervention group as compared to the control group (Table 2). In contrast, there were no significant differences between the two groups with regard to the annual changes in the skeletal traits of the FN. The results remained after adjustment for age at baseline and changes in weight and height (Table 2) as well as after adjustment for age at baseline and changes in fat and lean mass (data not shown). The mean annual increase in FN BMC was 13.1% in the intervention group compared to 10.8% in the control group (p = 0.53), and the corresponding values for periosteal diameter were 3.9% vs. 2.9% (p = 0.54) and for endosteal diameter 3.9% vs. 2.7% (p = 0.49).

**Table 2:** Baseline data and annual changes in anthropometry and femoral neck bone parameters.

	**Baseline**			**Annual changes**		**P-value**	**P-value**
	
	**Intervention**	**Control**	**p-value**	**Intervention**	**Control**	**unadjusted**	**adjusted**
Age (years)	7.7 ± 0.6	7.9 ± 0.6	0.08				
**Anthropometry**							
Weight (kg)	27.6 ± 5.5	27.4 ± 5.5	0.84	3.5 ± 2.2	3.2 ± 1.3	0.42	
Height (cm)	128 ± 5.2	129.1 ± 7.9	0.43	6 ± 1.2	5.7 ± 1.0	0.19	
Lean mass (kg)	19.8 ± 2.3	20.2 ± 2.8	0.42	2.2 ± 0.8	1.9 ± 0.6	**0.01**	
Fat mass (kg)	5.3 ± 3.9	5.2 ± 3.3	0.86	1.9 ± 1.5	1 ± 0.9	**< 0.001**	
**Bone mass of femoral neck**							
Bone mineral content (BMC; g)	2.63 ± 0.563	2.54 ± 0.497	0.39	0.266 ± 0.554	0.252 ± 0.236	0.87	0.86
Bone mineral density (BMD; g/cm^2^)	0.743 ± 0.089	0.701 ± 0.090	**0.04**	0.038 ± 0.071	0.045 ± 0.042	0.61	0.86
Volumetric bone mineral density (vBMD; g/cm^3^)	0.377 ± 0.044	0.359 ± 0.047	0.07	0.010 ± 0.037	0.012 ± 0.024	0.81	0.55
**Biomechanical calculations of femoral neck**							
Neck length (cm)	3.18 ± 0.401	3.25 ± 0.356	0.41	0.209 ± 0.348	0.237 ± 0.129	0.63	0.73
Periosteal width (cm)	2.46 ± 0.234	2.46 ± 0.225	0.89	0.086 ± 0.247	0.067 ± 0.113	0.64	0.68
Cross sectional area (CSA; cm^2^)	0.947 ± 0.156	0.889 ± 0.152	0.11	0.084 ± 0.180	0.091 ± 0.078	0.82	0.67
Section modulus (Z; cm^3^)	0.298 ± 0.090	0.272 ± 0.082	0.16	0.044 ± 0.088	0.049 ± 0.042	0.73	0.67
Cross-sectional moment of inertia (CSMI; cm^4^)	0.374 ± 0.159	0.338 ± 0.130	0.25	0.069 ± 0.167	0.074 ± 0.067	0.86	0.81
Endosteal width (cm)	2.30 ± 0.228	2.32 ± 0.224	0.74	0.078 ± 0.240	0.057 ± 0.114	0.59	0.62
Mean cortical thickness (cm)	0.076 ± 0.009	0.071 ± 0.009	**0.02**	0.004 ± 0.008	0.005 ± 0.005	0.44	0.97

Bone mass and biomechanical calculations of femoral neck in the intervention group (n = 53) and control group (n = 50). After exclusion of outliers, technical errors, and missing scans, anthropometrics were evaluated in 53 cases and 50 controls and femoral neck variables in 42 cases and 43 controls.

The data represent absolute values (means) ± SD. Comparison of annual changes in the intervention and control groups presented with the p-value unadjusted or adjusted for age at baseline and annual changes in weight and height. For BMD of femoral neck and mean cortical thickness, p-values were also adjusted in relation to the baseline values.

The weekly duration of physical activity during the study period did not correlate with the changes in the FN traits.

## Discussion

We did not find that the exercise program conferred any bone mineral or structural benefits on FN. Nor the weekly duration of training correlated with the gain in FN bone mass or structural parameters. This report is noteworthy, because it refutes the assumption of some researchers that the structural benefits of exercise will be missed if evaluations are limited to bone mineral accrual [10]. The previous reports of exercise induced benefits at the lumbar spine in this cohort [8,9,17] but not in the FN, is not surprising as weight-loaded skeletal regions with trabecular bone are more responsive to mechanical load than cortical regions [6,8,9,36].

The FN structural data in this report opposes some publications in growing children [14,37] but totally or partly supports others [15-17]. The discrepancies in the conclusions when comparing the trials could be due to differences in the design in the trials. The maturational level of the girls was different in the trials, and it is known that exercise induced skeletal benefits are most obvious in pre-menarcheal children [11,15,38,39]. The intensities and the proportions of the activities in the exercise programs were different, and it is known that high magnitude repetitive interventions confer more benefits than repeated endurance training [14,15,39,40]. The follow-up periods varied a fact that also could influence the outcome, as structural benefits may need a longer training period before reaching significance. That is, the study can't exclude that a different exercise program, for example a more intense training program or a program spanning a longer period, possibly could lead to benefits also in FN. As examples, tennis training has been reported to be associated with geometrical benefits [1,10] and soccer training for three years has in pre-pubertal boys been associated with obvious bone mass benefits also in FN [41].

The structural estimate of the FN by HSA may also differ from the three dimensional techniques, computed tomography (CT) and magnetic resonance imaging (MRI) [10,42], as the HSA is based on a two dimensional scanning technique [12]. In addition, no cited studies have objectively measured the level of physical activity, only relied on subjective estimate of training by questionnaires, and it is not certain that duration of exercise is the best method to quantify the level of physical activity. The higher spare time activities in the control group could also mask eventual beneficial osteogenic effects in the intervention group. Furthermore, it would have been beneficial if we could define the different type of spare time activities, not only the duration of the organized training activities. The population-based design of this trial is a strength, while the inability to perform individual randomization must be regarded as a weakness, as discussed in our previous reports [8,9]

The technical limitations of the HSA technique must also be highlighted. Inconsistent positioning of the limb or placement of the region of interest results in errors [12,13]. A small width error results in large errors in CSMI, as CSMI is proportional to the fourth power of the radius [5,43]. This is the reason why we excluded outliers as done in previous reports [12]. The cortical thickness and endosteal diameter are estimated after making assumptions of a homogenous porosity in the cortical shell, a homogenous cross-sectional shape and a fixed distribution of trabecular and cortical bone within the FN, assumptions not been tested in children. The estimation of the periosteal dimension are also derived from a two-dimensional image, but the skeleton can expand in other directions [10,44], may be not captured by the HSA. The quality of the resolution by the HSA analyses may also lead to small group differences being missed. In spite of these limitations in mind, HSA should be regarded as one method to focus the interest on not only on the amount of bone mineral but also FN structure, before three dimensional measuring techniques become general available in research.

It is also well known that differences in fat in the marrow or the soft tissue above, below or around the bones may affect the DXA bone variables [45]. However, our results and inferences remained after adjusting for age at baseline and changes in fat and lean mass.

## Conclusion

A one-year moderately intense school based exercise intervention program in pre-pubertal girls seems not to influence the by the DXA technique and HSA software estimated FN structure. Further studies are required to determine if an exercise program exceeding one year, a program with a higher intensity of training and a program in early peri-pubertal girls could be beneficial for the FN structure.

## Competing interests

The author(s) declare that they have no competing interests.

## Authors' contributions

Study design: MK and PG; Data collection: CL, JB and SSL; Statistical analyses and hip strength analyses and writing of the paper: GA, HA and MK. All authors read and approved the final manuscript

## Pre-publication history

The pre-publication history for this paper can be accessed here:


